# The Estrogenic Effect of Bisphenol A Disrupts Pancreatic β-Cell Function *In Vivo* and Induces Insulin Resistance

**DOI:** 10.1289/ehp.8451

**Published:** 2005-09-20

**Authors:** Paloma Alonso-Magdalena, Sumiko Morimoto, Cristina Ripoll, Esther Fuentes, Angel Nadal

**Affiliations:** 1 Instituto de Bioingeniería, Universidad Miguel Hernández de Elche, Alicante, Spain; 2 Departamento de Biología de la Reproducción, Instituto Nacional de Ciencias Médicas y Nutrición “Salvador Zubirán,” México City, México

**Keywords:** bisphenol A, diabetes, endocrine disruptors, estradiol, estrogen receptor, insulin, islet of Langerhans, nongenomic, xenoestrogens

## Abstract

The function of the pancreatic β-cell is the storage and release of insulin, the main hormone involved in blood glucose homeostasis. The results in this article show that the widespread environmental contaminant bisphenol-A (BPA) imitates 17β-estradiol (E_2_) effects *in vivo* on blood glucose homeostasis through genomic and nongenomic pathways. The exposure of adult mice to a single low dose (10 μg/kg) of either E_2_ or BPA induces a rapid decrease in glycemia that correlates with a rise of plasma insulin. Longer exposures to E_2_ and BPA induce an increase in pancreatic β-cell insulin content in an estrogen-receptor–dependent manner. This effect is visible after 2 days of treatment and starting at doses as low as 10 μg/kg/day. After 4 days of treatment with either E_2_ or BPA, these mice developed chronic hyperinsulinemia, and their glucose and insulin tolerance tests were altered. These experiments unveil the link between environmental estrogens and insulin resistance. Therefore, either abnormal levels of endogenous estrogens or environmental estrogen exposure enhances the risk of developing type 2 diabetes mellitus, hypertension, and dyslipidemia.

Insulin resistance is a crucial constituent of the metabolic syndrome, and its presence predicts type 2 diabetes and atherosclerotic cardiovascular disease (DeFronzo and Ferrannini 1991). In addition to insulin resistance, type 2 diabetes mellitus is also characterized by a progressive β-cell dysfunction. In most patients, both symptoms are present several years before the onset of hyperglycemia.

The incidence of diabetes has increased in the last decades, and at present it is reaching epidemic levels (177 million persons with diabetes in the world; World Health Organization 2005). The cornerstones of its development are related to modern lifestyle, principally, a lack of exercise and an unhealthy diet. Other pathologies whose incidence rose dramatically after World War II, such as cancer, reproductive impairment, and neurodegenerative diseases, are probably related to the increase of endocrine-disrupting chemicals (EDCs) in the environment (Colborn et al. 1996). However, an experimental link between EDCs and diabetes has not yet been established, although a connection at the epidemiologic level in humans has been recently proposed for dioxin, an environmental contaminant that acts through other than estrogen receptors (ERs) as an endocrine disruptor (Bertazzi et al. 2001; Rene and Bunce 2002).

A large number of EDCs act by mimicking the action of the sex hormone 17β-estradiol (E_2_) (Colborn et al. 1993). In most cases they bind to the classic ERs, ER-α and ER-β (McLachlan 2001; Newbold 2004), but they can also act through novel estrogen targets (Nadal et al. 2005). At physiologic levels, E_2_ is thought to be involved in maintaining normal insulin sensitivity and to be beneficial for β-cell function (Livingstone and Collison 2002; Louet et al. 2004). However, abnormal levels of E_2_ may promote insulin resistance (Livingstone and Collison 2002), similar to what occurs in normal puberty and pregnancy (Amiel et al. 1991; Hollingsworth 1983). Therefore, the exposure to an exogenous chemical acting as the natural hormone E_2_, but at an inappropriate concentration and during an improper time window, may enhance the risk of developing insulin resistance.

In spite of the many clinical studies that link sex steroids and actions of insulin, few studies have investigated the molecular basis of the interaction between E_2_, the pancreatic β-cell function, blood glucose homeostasis, and the development of diabetes. Pancreatic β-cells contain both types of ERs, ER-α and ER-β (Nadal et al. 2000). Although their functions are still greatly undetermined, ER-α and ER-β are involved in important aspects of the β-cell physiology (Nadal et al. 2004; Sutter-Dub 2002). These include protection against β-cell death caused by cytokines (Contreras et al. 2002) and, after a prolonged application, a beneficial effect on diabetes in mice expressing human islet amyloid peptide (Geisler et al. 2002). The involvement of ERs in lipid and glucose metabolism has been demonstrated in ER-α knockout mice that display increased adiposity, insulin resistance, and glucose intolerance (Heine et al. 2000). In addition, β-cells have the nonclassical membrane ER (ncmER) that triggers rapid effects (Nadal et al. 1998, 2000; Quesada et al. 2002). Recently, a similar receptor has been found in *Drosophila* (Srivastava et al. 2005). E_2_ rapidly potentiates β-cell signaling systems and insulin release via this ncmER, an effect that is mimicked by EDCs, including bisphenol A (BPA) (Nadal et al. 2004). BPA is one of the most common chemicals that behaves as an endocrine disruptor. It was the first synthetic estrogen without a steroid structure (Dodds and Lawson 1936), but because of its properties as a cross-linking chemical, BPA was widely chosen by the chemical industry to produce plastic polymers, mainly poly-carbonates. Nowadays, it is used in the manufacture of barrier coatings for the inner surfaces of food and beverage cans. High concentrations of BPA have been detected in food and water extracted from autoclaved cans (Brotons et al. 1995). BPA is one of the highest-volume chemicals produced in the world, and its exposure is widespread: it has been found in 95% of the urine samples from people in the United States and to a similar extent is found in human blood, as well (Ikezuki et al. 2002; vom Saal and Hughes 2005). The European Commission’s Scientific Committee on Food (ECSCF 2002) reported a tolerable daily intake (TDI) of 10 μg/kg/day. However, the U.S. Environmental Protection Agency (U.S. EPA) considers 50 μg/kg/day the reference dose based on the lowest observed adverse effect level (LOAEL) of 50 mg/kg/day, according to studies performed in the 1980s (vom Saal and Hughes 2005). *In vivo* studies using much lower doses of BPA than the LOAEL have shown that it affects sexual maturation (Howdeshell et al. 1999), induces a decrease in daily sperm count and fertility (vom Saal et al. 1998), disrupts chromosome alignment (Hunt et al. 2003), and affects synaptogenesis (McLusky et al. 2005). In spite of this evidence, there is an ongoing debate with the chemical industry that is still skeptical about the risk of low doses of BPA (Bisphenol A Global Industry Group 2005).

In this article we show that the exposure of adult mice to BPA, at doses about 1,000-fold less than the LOAEL established by the U.S. EPA, alters blood glucose homeostasis *in vivo*. It rapidly increases plasma insulin, altering blood glucose concentration through a nonclassical estrogen pathway. Longer exposure increases β-cell insulin content, an effect that involves classic ERs. These longer exposures generate chronic hyperinsulinemia in the fed state and peripheral insulin resistance.

## Materials and Methods

### Materials.

We obtained ICI182,780 (ICI) from Tocris Cookson Ltd. (Avonmouth, UK), tocopherol-stripped corn oil from MP Biomedicals, LLC (Solon, OH, USA), and soluble insulin from Humulina Regular (Lilly, Madrid, Spain). Other substances were obtained from Sigma (Madrid, Spain).

### Animals.

We used Swiss albino OF1 male mice (8–10 weeks of age) throughout this study. All animals were kept under standard housing conditions and were treated humanely and with regard for alleviation of suffering. An internal animal care and use committee reviewed and approved the method used.

### Treatment.

Stimuli (E_2_ and BPA) were dissolved in tocopherol-stripped corn oil and administered subcutaneously at various concentrations. The amount of vehicle was kept constant at 100 μL. In long-term experiments, animals were injected twice per day, at 0900 hr and 2000 hr, with 50 μg/kg or 5 μg/kg of test compound. Two injections of 50 μg/kg/day during 4 days gave a plasma concentration of E_2_ similar to that found in late pregnancy (Song et al. 2001). In ICI experiments, animals were injected intraperitoneally with a single dose of 500 μg/kg/day for 3–4 days, always at 0800 hr.

### Glycemia determination.

We determined glucose in blood obtained from the tail vein using an Accu-check compact glucometer (Roche Diagnostic GmbH, Mannheim, Germany).

### Insulin secretion and content.

To measure plasma insulin, mice were anesthetized with 50 mg/kg body weight sodium pentobarbital. Blood (~ 1 mL) was obtained by cardiac puncture with a syringe containing 24 mM EDTA. We determined the levels of plasma insulin by enzyme-linked immunosorbent assay (ELISA) using a mouse insulin assay kit from Mercodia AB (Uppsala, Sweden).

To measure insulin release from isolated islets, mice were killed by cervical dislocation, and pancreatic islets of Langerhans were isolated by collagenase digestion as described previously (Morimoto et al. 2001). Islets were washed twice with a buffer solution containing 20 mM HEPES, 115 mM NaCl, 5 mM NaHCO_3_, 5 mM KCl, 2.6 mM CaCl_2_, 1.2 mM KH_2_PO_4_, 1.2 mM MgSO_4_, 3 mM d-glucose, and 1% bovine serum albumin (pH 7.4). Groups of 10 islets were then incubated in 1 mL of this buffer in the presence of 3, 7, and 16 mM glucose. After 1 hr, the medium was collected, and insulin was measured in duplicate samples by radioimmunoassay using a Coat-a-Count kit (DPC, Los Angeles, CA, USA).

The insulin content was determined in islets isolated as described above. Islets were grouped in batches of 10 and incubated overnight in an ethanol/HCl buffer at 4°C. At the end of the incubation period, the buffer was removed and studied for insulin content using radioimmunoassay with a Coat-a-Count kit (DPC). Protein determination was performed by the Bradford dye method.

### Immunocytochemistry and insulin content.

Islets isolated as above were dispersed into single cells as previously described (Nadal et al. 1998). Briefly, islets were disaggregated into single cells with trypsin. Cells were then centrifuged and resuspended in Hank’s modified medium supplemented with 200 U/mL penicillin, 0.2 mg/mL streptomycin, 5 mM glucose, and 1% fatty acid–free albumin, pH 7.4. They were then plated on 24-well tissue culture plates. After 30 min, cells were washed with phosphate-buffered saline (PBS) and fixed with Bouin’s solution for 5 min. Then, they were dehydrated with 30, 50, and 70% ethanol, 3 min each, and then washed with PBS. After washing, these cells were first incubated with a monoclonal anti-insulin antibody (1:200 dilution; Sigma, Madrid, Spain) for 2 hr and then for 1 hr with a secondary antibody, anti-mouse IgG-conjugated fluorescein isothiocyanate (IgG-FITC; 1:200 dilution; Sigma), both at room temperature. Cells were washed with PBS overnight. Images were acquired with a confocal microscope Zeiss Pascal 5 using a Zeiss 20× objective (numerical aperture = 0.5) and analyzed using LSM Zeiss software (Zeiss, Jena, Germany). We measured immunofluorescence intensity in random fields. The results were expressed as average pixel intensity and normalized with respect to vehicle-treated animals. Pixel intensity was normalized with respect to the mean value of the pixel intensity of vehicle-treated animals at the corresponding days.

### Glucose and insulin tolerance tests.

For glucose tolerance tests, animals were fasted overnight for 12 hr, and blood samples were obtained from the tail vein. Animals were then injected intraperitoneally with 2 g/kg body weight of glucose, and blood samples were taken at the indicated intervals.

For insulin tolerance tests, fed animals were used. Animals were injected intra-peritoneally with 0.75 IU/kg body weight of soluble insulin. Blood glucose was measured in each sample using an Accu-check compact glucometer (Roche).

### Statistical analysis.

Data are expressed as mean ± SE. Pairwise comparisons were made using Student’s *t*-test. A probability level < 0.05 was considered statistically significant.

## Results

### E_2_ and BPA rapidly alter glycemia and insulinemia.

**Figure 1:**
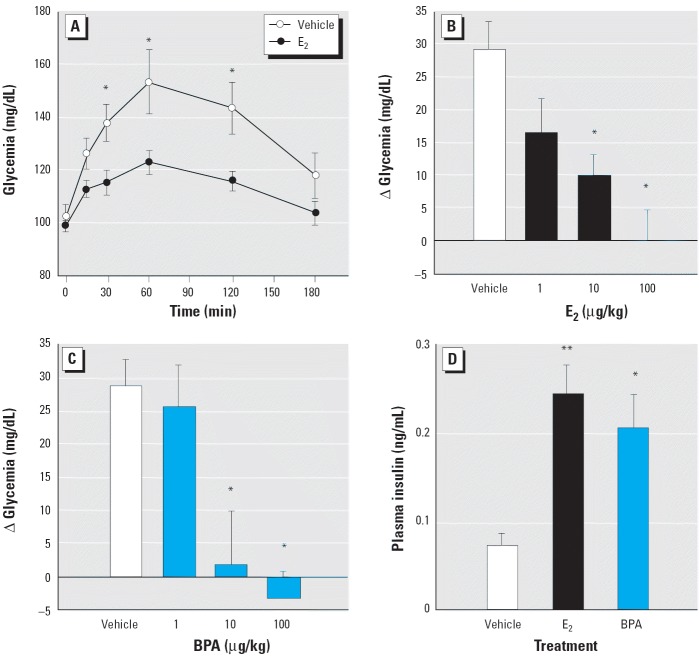
Rapid change in blood glucose levels with E_2_ and BPA compared with tocopherol-free corn oil (vehicle). (*A*) Measurement of blood glucose concentration in animals fasted for 12 hr, injected with 100 μL vehicle or 10 μg/kg body weight E_2_ (*n* = 6–14 mice); *p < 0.05. (*B*) Increment of glycemia 30 min after the injection of vehicle or E_2_ (*n* = 7–16); *p < 0.05 compared with vehicle. (*C*) Increment of glycemia 30 min after the injection of vehicle or BPA (*n* = 4–10); *p < 0.05 compared with vehicle. (*D*) Circulating plasma insulin in fasted (12 hr) animals 30 min after the injection of vehicle, 10 μg/kg E_2_ or 10 μg/kg BPA (*n* = 8–16); *p = 0.024, and **p = 0.004 compared with vehicle. Error bars indicate SE.

**Figure 2:**
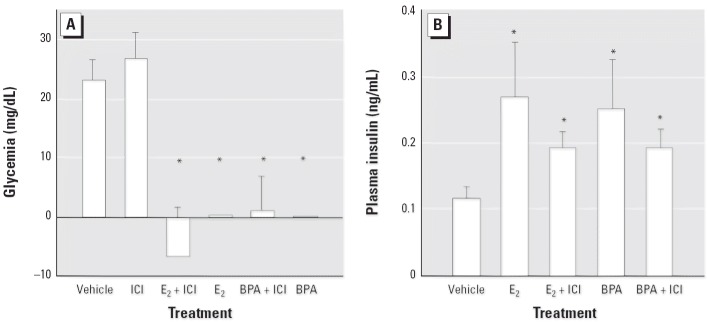
Increment of glycemia (*A*) and plasma insulin (*B*) 30 min after the injection of vehicle, 10 μg/kg E_2_, or 10 μg/kg BPA in animals with or without treatment with ICI (500 μg/kg/day) for 3 days. In (*A*), *n* = 4–12; *p < 0.002 compared with ICI or vehicle. In (*B*), *n* = 4–7; *p < 0.035 compared with vehicle.

*In vitro*, both E_2_ and BPA alter the function of pancreatic β-cells through the ncmER (Nadal et al. 1998, 2000; Quesada et al. 2002; Ropero et al. 2002). *In vivo*, the administration of 10 μg/kg E_2_ to adult male mice resulted in a significant decrease of glycemia measured at 30, 60, and 120 min after the injection, compared to the increase of blood glucose produced by the fatty acids contained in the vehicle injection (tocopherol-stripped corn oil) (Figure 1A). The administration of 1, 10, and 100 μg/kg E_2_ evoked a clear dose-dependent decrease in the rise of glycemia 30 min after the E_2_ injection (Figure 1B). This effect is mimicked by equal doses of the environmental estrogen BPA (Figure 1C). Thirty minutes after injection, this decrease in blood glucose is parallel to an increase in plasma insulin (Figure 1D) of 3.20 ± 0.45-fold for E_2_-treated mice and 2.76 ± 0.5-fold for BPA-treated mice. Rapid effects elicited by E_2_ and BPA in islets of Langerhans *in vitro* are initiated after they bind at the ncmER that is insensitive to the pure anti-estrogen ICI (Nadal et al. 2004). This anti-estrogen was described as blocking classic ER-mediated actions *in vivo* (Johnson et al. 2003; Perez-Martin et al. 2003). To evaluate whether the E_2_ and BPA actions described above require an ER with a sensitivity to the pure antiestrogen similar to a classical ER, we undertook experiments using mice treated with 500 μg/kg/day ICI administered intraperitoneally for 3 days (Johnson et al. 2003). As shown in Figure 2A, ICI had no effect on the E_2_ and BPA-dependent blood glucose decrease nor on the E_2_- and BPA-dependent increase of plasma insulin (Figure 2B).

Therefore, both E_2_ and BPA rapidly change glycemia most likely by inducing an hypersecretion of insulin through a non-classical ER-mediated mechanism that may involve the ncmER previously described in these cells (Nadal et al. 2000, 2004).

### E_2_ and BPA increase β-cell insulin content.

**Figure 3:**
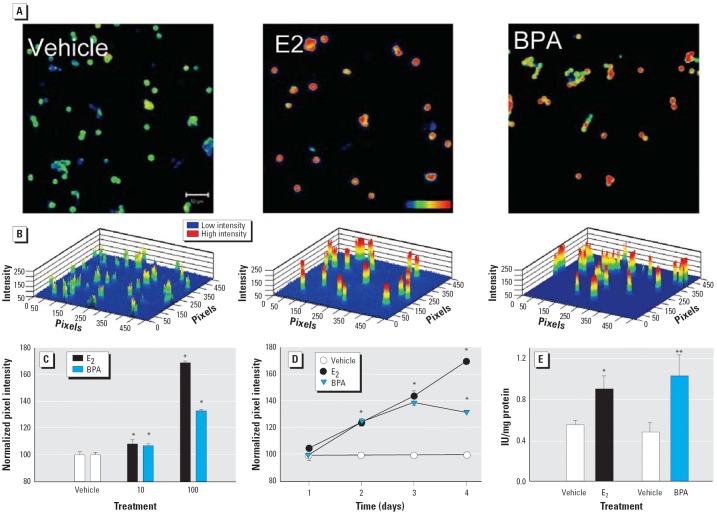
Insulin content in β-cells from E_2_- and BPA-treated mice. (*A*) Immunofluorescent staining of insulin in cells from mice treated with vehicle, 100 μg/kg/day E_2_, or 100 μg/kg/day BPA for 4 days. Bar = 50 μm; blue indicates low fluorescence intensity, and red indicates high intensity. (*B*) Three-dimensional graphs of cells in (*A*), showing the pixel intensity [0 (low) to 256 pixels (high)]. (*C*) Quantification of insulin content using confocal microscopy of β-cells from mice treated with vehicle, E_2_, or BPA for 4 days at either 10 or 100 μg/kg/day, shown as normalized pixel intensity. Each point represents the mean of at least 1,000 cells from three mice; *p < 0.003 compared with vehicle. (*D*) Time course indicating E_2_ and BPA action in β-cells. Each point represents the mean of at least 1,000 single cells obtained from two mice; *p < 10^–5^ compared with vehicle. (*E*) Insulin content of islets obtained from mice treated with vehicle, 100 μg/kg/day E_2_ (*n* = 6; *p = 0.014), or 100 μg/kg/day BPA for 4 days (*n* = 6; **p = 0.04). All error bars indicate SE.

Adult male mice were injected twice a day with the vehicle, E_2_ or BPA for 4 days. Afterward, insulin content was measured in individual cells by immunocytochemistry. Figure 3A shows confocal images of β-cells obtained from mice treated 4 days with vehicle, 100 μg/kg/day E_2_, or 100 μg/kg/day BPA. Figure 3B illustrates a three-dimensional reconstruction of images in Figure 3A showing a pixel intensity scale from 0 to 256 pixels. The images and the three-dimensional graphs illustrate that β-cells from animals treated with E_2_ and BPA presented higher staining than those treated with the vehicle and thus higher insulin content in every cell. After 4 days of treatment, the insulin increase was already manifested at doses of 10 μg/kg/day of either E_2_ or BPA (Figure 3C). Nonetheless, this effect was small and 100 μg/kg/day was needed to produce a potent increase in insulin content (Figure 3C). A treatment of 100 μg/kg/day E_2_ for 4 days gives an E_2_ plasma concentration similar to that found in late pregnancy (Song et al. 2001). Therefore, the chronic action of E_2_ and BPA was manifested at higher concentrations than was required for the acute effect. On the basis of this response, we use 100 μg/kg/day as the paradigmatic concentration in long-term experiments.

Time-course experiments demonstrated that the onset of the increase in insulin content occurs after 24–48 hr of treatment with either E_2_ or BPA (Figure 3D). The experiment in Figure 3D shows that most single β-cells increased their insulin content. The experiment in Figure 3D shows that after 4 days of treatment, the insulin content is higher in E_2_ and BPA-treated mice. The radioimmunoassay analysis of insulin content performed 4 days after the treatment with 100 μg/kg/day of either E_2_ or BPA produced similar results as those presented with immunocytochemistry (Figure 3E). These results demonstrate that the insulin content was increased by both the natural hormone and the environmental estrogen to a similar extent.

**Figure 4:**
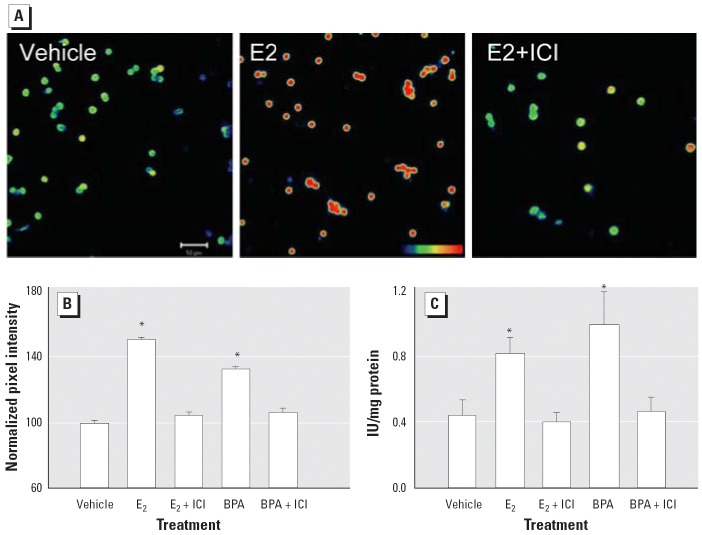
Insulin content in β-cells from E_2_- and BPA-treated mice with and without treatment with the pure antiestrogen ICI. (*A*) Immunofluorescent staining for insulin in mice treated with vehicle, 100 μg/kg/day E_2_, or E_2_ plus 500 μg/kg/day ICI for 4 days. Bar = 50 μm; blue indicates low fluorescence, and red indicates high fluorescence. (*B*) Quantification of insulin content using confocal microscopy of β-cells from mice treated with vehicle, 100 μg/kg/day E_2_ or BPA, or 100 μg/kg/day E_2_ or BPA plus 500 μg/kg/day ICI. Each point represents the mean of at least 2,000 individual cells from four mice; *p < 10^–10^. (*C*) Insulin content obtained by radioimmunoassay (*n* = 6); *p < 0.05, comparing E_2_ with E_2_+ICI and BPA with BPA+ICI. All error bars indicate SE.

To study the involvement of classical ERs in the regulation of E_2_- and BPA-induced insulin expression, we used mice treated with the antiestrogen ICI, as described for the experiment presented in Figure 2. Animals treated with 100 μg/kg/day E_2_ for 4 days presented higher levels of insulin immunoreactivity than the control (Figure 4A). In animals treated with ICI, E_2_ had no effect (Figure 4A). The action of the pure antiestrogen is manifested to the same extent with BPA, as demonstrated by immunocytochemistry (Figure 4B) and radioimmunoassay (Figure 4C). The ability of the pure anti-estrogen to completely block the effect of both E_2_ and BPA indicates that the action performed on insulin content is mediated by a classical ER.

### E_2_ and BPA administration provokes hyperinsulinemia and insulin resistance.

**Figure 5:**
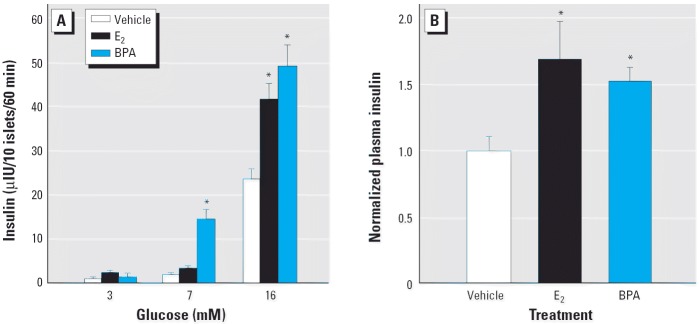
Insulin secretion *in vitro* and *in vivo*. (*A*) Glucose-induced insulin secretion from isolated islets at 3, 7, and 16 mM glucose, from mice treated with vehicle, 100 μg/kg/day E_2_, or 100 μg/kg/day BPA for 4 days (*n* = 4–6 animals per group); *p < 0.005, compared with vehicle. (*B*) Normalized plasma insulin with respect to the plasma concentration in mice treated with vehicle, 100 μg/kg/day E_2_ or 100 μg/kg/day BPA for 4 days (*n* = 5–10 mice per group); *p < 0.0075 compared with vehicle. In the E_2_ group, circulating insulin levels were 1.53 ± 0.25 ng/mL for vehicle-treated mice and 2.58 ± 0.42 ng/mL for E_2_-treated mice (*n* = 5; *p* = 0.038); in the BPA group, circulating insulin levels were 1.02 ± 0.14 ng/mL for the vehicle-treated mice and 1.56 ± 0.11 ng/mL for those treated with BPA (*n* = 7; *p* = 0.005). All error bars indicate SE. Both vehicle groups were combined; the data are normalized with respect to the vehicle value from each group.

An increment in the insulin content of every β-cell within an islet causes it to release a higher amount of insulin every time it is stimulated (Marban et al. 1989). This occurs, for instance, during pregnancy, when islets adapt to deal with peripheral insulin resistance (Costrini and Kalkhoff 1971; Faure et al. 1985). In our system (Figure 5A), we show that, at high glucose concentrations (16 mM), islets from mice treated with 100 μg/kg/day E_2_ for 4 days secrete 1.54 ± 0.12-fold more insulin than those from mice treated with the vehicle, and the same occurs in islets from mice treated with BPA. In the latter case, differences are significant even at lower glucose concentrations (7 mM). This effect has been recently described *in vitro* (Adachi et al. 2005). Therefore, if E_2_- and BPA-treated mice have higher insulin content, they should release more insulin than untreated mice, and accordingly, these animals should be hyperinsulinemic in the fed state. This is shown in Figure 5B: 4 days after the E_2_ treatment, fed mice had blood insulin levels 1.7-fold higher than those of vehicle-treated mice. Blood glucose levels were 186 ± 5 mg/dL in vehicle-treated mice and 170 ± 6 mg/dL in E_2_-treated mice (*p* = 0.065, not significant). Plasma insulin levels for the group treated with BPA were 1.5-fold higher than those for the vehicle-treated mice; however, their blood glucose levels were 153 ± 4 mg/dL for the vehicle (*n* = 5) and 154 ± 7 mg/dL for BPA (*n* = 7; *p* = 0.93). This experiment indicates that, in the E_2_-treated animals, there was mild insulin resistance, because there are 1.7-fold higher circulating insulin levels but a decrease of blood glucose, although it is not significant. This effect is remarkably manifested with BPA; in this case, plasma insulin levels are 1.53-fold higher and blood glucose concentration does not vary, a clear symptom of insulin resistance.

**Figure 6:**
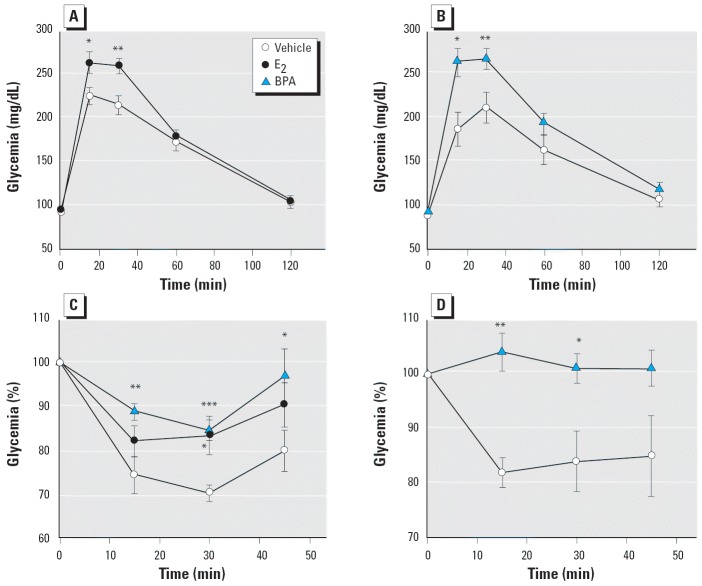
E_2_ and BPA alter glucose tolerance and induce insulin resistance. (*A*) Glucose tolerance test in mice treated with vehicle or 100 μg/kg/day E_2_ for 4 days; (*n* = 16); *p = 0.02, and **p = 0.003. (*B*) Same experiment as in (*A*) but with animals treated with vehicle or 100 μg/kg/day BPA (*n* = 8); *p = 0.017, and **p = 0.009. (*C*) Insulin tolerance test in awake, fed mice previously treated with vehicle, 100 μg/kg/day E_2_, or 100 μg/kg/day BPA (*n* = 9); *p < 0.04, **p = 0.007, and ***p = 0.0002 compared with vehicle. (*D*) Experiment as in (*C*) but using an oral intake of either vehicle or 100 μg/kg/day BPA (*n* = 5); *p = 0.026, and **p = 0.0012. All error bars indicate SE.

To prove that glucose tolerance is altered, we performed an intraperitoneal glucose tolerance test in fasted (12 hr) mice. Contrary to vehicle-treated mice, blood glucose increased to a higher level in E_2_-treated (Figure 6A) and BPA-treated mice (Figure 6B). The higher increase in blood glucose manifested 15 and 30 min after a glucose challenge. We found that the area under the curve (milligrams per deciliter-minute) was 162 ± 6 (vehicle, *n* = 24), 181 ± 5 (E_2_, *n* = 16, *p* = 0.025 vs. vehicle), and 190 ± 8 (BPA, *n* = 8, *p* = 0.028 vs. vehicle). These results indicate that an impaired glucose tolerance was present in E_2_-treated mice and in those treated with BPA. When an insulin tolerance test (intraperitoneal injection of 0.75 IU/kg body weight soluble insulin) was performed in fed mice, a significantly reduced hypoglycemic response was observed in both E_2_- and BPA-treated mice (Figure 6C). This insulin intolerance also manifested in animals given an oral dose of 100 μg/kg/day BPA for 4 days. In this case, the BPA was dissolved in tocopherol-stripped corn oil and delivered through a pipette placed into the animal’s mouth (Howdeshell et al. 1999) (Figure 6D). All these results indicate that E_2_-treated animals and, in a remarkably similar manner, BPA-treated animals develop insulin resistance without any changes in glycemia or weight (35–40 g, with no significant differences between the diverse treatments applied).

## Discussion

The results presented in this article demonstrate a link between estrogenic endocrine disruptors and insulin resistance. We have shown that BPA mimics E_2_ effects on blood glucose homeostasis through two different pathways. A nonclassical pathway produces a rapid increase in plasma insulin and a decrease in blood glucose. This is unaffected by the anti-estrogen ICI and is most likely initiated by the ncmER already described in islet cells (Alonso-Magdalena et al. 2005; Nadal et al. 2000; Quesada et al. 2002; Ropero et al. 2002). Nonetheless, other estrogen and xenoestrogen actions initiated at the plasma membrane are mediated via classical ERs (Losel et al. 2003; Wozniak et al. 2005). The activation of this alternative pathway occurs at low doses of both E_2_ and BPA, becoming significant at 10 μg/kg. When animals were treated with either the natural hormone or BPA for a longer period of time, there was an increase in the pancreatic insulin content. This effect was completely blocked by ICI, suggesting that a classical ER is involved. This implies that the action described here for E_2_ and BPA can be extrapolated to other estrogenic EDCs. Any EDC having an estrogenic effect through classical ERs can be a candidate to induce insulin overexpression. This chronic treatment induced insulin resistance.

Type 2 diabetes mellitus is characterized by insulin resistance, which results in lower levels of blood glucose uptake into target tissues. Consequently, blood glucose levels increase and more insulin is released, producing hyperinsulinemia, which manifests early in type 2 diabetes. In addition, several studies have demonstrated that the hypersecretion of insulin is a primary defect of type 2 diabetes and that insulin resistance develops secondarily to the chronic hyperinsulinemia (Charollais et al. 2000; DeFronzo 1997; Devedjian et al. 2000; McGurry 1992). Indeed, the persistence of chronic physiologic euglycemic hyperinsulinemia for 3–5 days can induce severe insulin resistance in healthy subjects with normal glucose tolerance (Del Prato et al. 1994). Furthermore, patients with insulinoma display a correlation between hyperinsulinemia and insulin resistance (Pontiroli et al. 1992; Skrha et al. 1996). When insulin is overexpressed, a chronic hyperinsulinemia manifests, as shown in transgenic mice that overexpressed the insulin gene (Marban et al. 1989). Remarkably, these transgenic mice developed insulin resistance. Here we have shown that 4 days of treatment with either E_2_ or BPA induces an increase in β-cell insulin content; these mice were hyper-insulinemic and presented altered glucose and insulin tolerance tests. These alterations may result from a direct effect of E_2_ and BPA on β-cell insulin content, or a compensatory response resulting from the insulin resistance noted in these animals, or both.

We have demonstrated that after 4 days of treatment, isolated islets respond more vigorously to glucose (Figure 5A), likely because their insulin content is higher (Marban et al. 1989). This phenomenon may be responsible for the chronic hyperinsulinemia that manifested in the fed state (Figure 5B). In addition, the altered glucose and insulin tolerance test results were consistent with the fact that these mice had developed insulin resistance. Therefore, our results suggest that the sustained hyperinsulinemia produced by E_2_ and BPA affects peripheral tissues, producing insulin resistance, most likely by down-regulation of insulin receptor number and function. However, the extent of the insulin resistance with this treatment is not enough to induce hyperglycemia in the fasted state.

The assumption described above does not rule out a direct effect of both E_2_ and BPA on peripheral tissue. BPA produces down-regulation of glucose transporters in adipocytes (Sakurai et al. 2004), an action that may induce insulin resistance. Moreover, BPA combined with insulin favors the conversion of fibroblasts to adipocytes (Masuno et al. 2002), enhancing the risk of obesity, a metabolic disorder that has been related to endocrine disruptors in the last years (Heindel 2003; Mead 2004). Hence, the direct effect of BPA on peripheral tissue might also be of importance to developing insulin resistance.

At present, there is a debate about determining the safe levels of BPA exposure and whether there is a need for a new risk assessment (vom Saal and Hughes 2005). The U.S. EPA considers 50 μg/kg/day as the reference dose based on a LOAEL of 50 mg/kg/day, according to studies performed in the 1980s (vom Saal and Hughes 2005). The ECSCF (2002) reported a TDI of 10 μg/kg/day. The results presented here show a rapid nongenomic effect at a dose of 10 μg/kg/day, five times lower than the reference dose established by the U.S. EPA and equal to the TDI reported by the ECSCF. This dose of BPA results in levels of parent (unconjugated) BPA in blood of 3–4 nM after 30 min and maintained 24 hr later (Zalko et al. 2003), below the level reported in blood from human fetuses at parturition (Schonfelder et al. 2002). This low dose of BPA produces a 2.5-fold increase in plasma insulin and a 20% decrease in blood glucose levels, 30 min after its application. Remarkably, a low dose of 10 μg/kg/day was able to slightly change insulin content as well. A higher dose of 100 μg/kg/day dramatically increased pancreatic insulin content after only 4 days of exposure. Moreover, this treatment, delivering BPA either via injection or through oral intake, induced insulin resistance. This dose is only twice the reference dose recommended by the U.S. EPA and 10 times higher than the TDI recommended by the ECSCF.

The present study demonstrates a connection between BPA and insulin resistance at doses much lower than the LOAEL used up to now (50 mg/kg/day), and therefore it is strong evidence supporting a review of the risk assessment concerning BPA.
